# Variants of *PLCXD3* are not associated with variant or sporadic Creutzfeldt-Jakob disease in a large international study

**DOI:** 10.1186/s12881-016-0278-2

**Published:** 2016-04-07

**Authors:** Rubika Balendra, James Uphill, Claire Collinson, Ronald Druyeh, Gary Adamson, Holger Hummerich, Inga Zerr, Pierluigi Gambetti, John Collinge, Simon Mead

**Affiliations:** MRC Prion Unit and Department of Neurodegenerative Disease, UCL Institute of Neurology, Queen Square, London, WC1N 3BG UK; Clinical Dementia Center, Department of Neurology, Georg-August University Göttingen, Göttingen, Germany; German Center for Neurodegenerative Diseases, Gottingen, Germany; Department of Pathology, Case Western Reserve University, Cleveland, OH 44060 USA

**Keywords:** Human prion diseases, Creutzfeldt-Jakob disease, *PLCXD3*

## Abstract

**Background:**

Human prion diseases are relentlessly progressive neurodegenerative disorders which include sporadic Creutzfeldt-Jakob disease (sCJD) and variant CJD (vCJD). Aside from variants of the prion protein gene (*PRNP*) replicated association at genome-wide levels of significance has proven elusive. A recent association study identified variants in or near to the *PLCXD3* gene locus as strong disease risk factors in multiple human prion diseases. This study claimed the first non-*PRNP* locus to be highly significantly associated with prion disease in genomic studies.

**Methods:**

A sub-study of a genome-wide association study with imputation aiming to replicate the finding at *PLCXD3* including 129 vCJD and 2500 sCJD samples. Whole exome sequencing to identify rare coding variants of *PLCXD3*.

**Results:**

Imputation of relevant polymorphisms was accurate based on wet genotyping of a sample. We found no supportive evidence that *PLCXD3* variants are associated with disease.

**Conclusion:**

The marked discordance in vCJD genotype frequencies between studies, despite extensive overlap in vCJD cases, and the finding of Hardy-Weinberg disequilibrium in the original study, suggests possible reasons for the discrepancies between studies.

## Background

Prion diseases are transmissible and fatal neurodegenerative disorders, affecting both humans and animals [1]. The human prion diseases comprise Creutzfeldt-Jakob disease (CJD), kuru, proteinase sensitive prionopathy, Gerstmann-Sträussler-Scheinker disease (GSS), fatal familial insomnia, and PrP Systemic Amyloidosis. 10–15 % of all human prion disease is inherited as a germline trait, caused by coding mutation of *PRNP.* Based on recent GWAS, the major common genetic determinant of sporadic or acquired prion disease risk is a missense polymorphism at codon 129 of *PRNP*, encoding for the cellular form of the prion protein, PrP^c ^[2].

Recently a re-analysis of a previously published GWAS [3] consisting of 85 vCJD cases and 1481 control individuals found that after *PRNP*, the region most significantly associated with disease was at the *PLCXD3* gene locus. Resequencing three intronic SNPs near the splice junction of intron 1 and exon 2 of this gene in 109 sCJD and 120 vCJD cases revealed that two of these SNPs showed marked Hardy-Weinberg disequilibrium and were highly significantly associated with disease compared to publicly available controls [4]. As this effect was extremely strong, we sought to replicate the finding in a further cohort of human prion disease cases to find out whether this gene was associated with increased disease risk and investigate any effects on clinical phenotype.

## Methods

### Summary and samples

We conducted a gene-specific study as a component of GWAS with imputation comprising 2500 sCJD cases, 129 vCJD cases and 10,548 control individuals using established methods for quality control and statistical analysis (see below). Patients with a diagnosis of sporadic CJD, of UK or Northern European residence, were recruited by the National Prion Clinic (NPC), London, the National CJD Research and Surveillance Unit, Edinburgh and other referrers in the UK in the period from 1995 to 2012. In the UK 60 % had pathologically confirmed sCJD, the remainder had a diagnosis of probable sCJD according to the published WHO criteria with a high specificity (http://www.cjd.ed.ac.uk/documents/criteria.pdf). Median age of disease onset was 65 years. In Germany and USA, all cases were pathologically confirmed. Probable or definite variant CJD patients were diagnosed according to established criteria (http://www.cjd.ed.ac.uk/documents/criteria.pdf). Due to the very small number of vCJD patients in the UK and worldwide (http://www.who.int/mediacentre/factsheets/fs180), and because of an effective system of sample sharing between Units in the UK, we can be confident that the vCJD cases in our study included the vast majority if not all 85 cases in the Bishop et al. study. Mean age of onset of disease in this cohort of patients was 30 years.

We calculated *p*-values of association for all SNPs located within *PLCXD3* and within 50 kilobases of the gene, including for the three SNPs sequenced in the previous study, rs545358, rs319013 and rs76547469, the former two of which were reported as having allele and genotype frequencies significantly different to publicly available European control populations [4] using SNPTESTv2.5β [5]. As genotype data in our GWAS was imputed we confirmed that imputation was accurate with both allele discrimination PCR probes and Sanger sequencing. To further ascertain mutations in *PLCXD3* we reviewed next generation whole exome sequencing data from 249 sCJD cases, 98 vCJD cases, 29 iatrogenic CJD cases and 665 non-prion neurodegenerative disease control individuals to identify non-synonymous variants in the gene (see below).

### Genome wide association study

Seven-hundred and thirty three UK sCJD cases, 818 German sCJD cases, 951 US sCJD cases, 129 vCJD cases, 5020 UK Wellcome Trust Case—control Consortium control individuals, 2691 German KORA control individuals and 2837 US control individuals were included. Samples were genotyped on Illumina 550 K, 660 K, Illumina Human 1.2 M-Duo Custom (for WTCCC2 controls) OmniExpress, Omni2.5 M or Omni5M arrays [6]. Prior to imputation, samples were excluded if they had less than a 98 % call rate. SNPs were excluded with a missingness greater than 1 %, minor allele frequency less than 1 % and Hardy—Weinberg disequilibrium in controls <1 × 10^−3^. Phasing and subsequent imputation were performed using SHAPEITv2 and Imputev2.3.0 employing the 1000 Genomes Phase 1 integrated variant set [7]. SNPTESTv2.5β was used for statistical association testing. This was performed using the frequentist score method, which takes genotype uncertainty into account, whilst applying an additive genetic model. Four population covariates from a principle components analysis (using PLINK multidimensional scaling function) were included (resulting in lambda = 1.06). Uncorrected *P* values are shown.

All samples were provided with informed consent for research genetic studies with approval of local research ethics committees. The study was approved by the London Harrow Research Ethics Committee.

### Confirmation of imputed genotypes with allele discrimination probes and Sanger sequencing

Allele specific probes and primers were used to confirm imputed genotypes at rs545358 and rs319013 in 176 sCJD and 119 vCJD cases. Sanger sequencing was used to further confirm genotypes across these SNPs for 46 sCJD and 47 vCJD cases. For PCR amplification the forward primer 5’-cacccataaggaaagccaat-3’and the reverse primer 5’-gggtctctgggcttggt-3’ were used and for sequencing the reverse primer reported in the previous study, 5’-catttccgcatgagcttttt-3’, was used [4].

### Whole exome sequencing

Two-hundred and forty nine sCJD exomes, 98 vCJD exomes, 29 iatrogenic CJD exomes and 665 control exomes (consisting of Alzheimer’s disease, frontotemporal dementia, Huntington’s disease phenocopy and glaucoma cases) were captured with SureSelect [8] and HaloPlex [9] (Agilent) kits. Exomes were sequenced on the Illumina HiSeq2000 platform with 100 bp paired-end runs. Sequences were aligned to the human reference genome using Novoalign software [10]. The Genome Analysis Toolkit (GATK) Unified Genotyper [11, 12] was used for SNP and indel calling then sequences were recalibrated with the GATK Variant Recalibrator [13] and variants annotated with ANNOVAR [14]. Variants were annotated for location, function, including whether they led to splice site changes, frameshift or stop codon mutations, for SIFT (sorting intolerant from tolerant) [15] and POLYPHEN (polymorphism phenotyping) [16] values, which predict the functional effect of a variant, and for whether they were novel, rare or somewhat rare based upon frequency in the control dataset of 0 %, <0.2 % and <0.5 % respectively. Variants were filtered out from the analysis if they were synonymous, read depth was <8, the variant had not been sequenced in >30 % of samples or minor allele frequencies of the variant in control populations from Exome Variant Server (http://evs.gs.washington.edu/EVS) and/or 1000 Genomes (http://www.ncbi.nlm.nih.gov/variation/tools/1000genomes/) was >10 %. For heterozygous calls a minimum of 18 % of reads were non-reference.

For statistical analysis, allelic Fisher’s Exact tests were performed for single variants associated with disease in cases compared to controls. In addition gene based tests were performed using a binomial probability test looking at all non-synonymous variants within a gene in cases compared to controls. Statistical analyses were performed using R statistical package version 3.0.2.

In particular we also looked for novel variants not found in any control populations. The control populations we referred to were the Wellcome Trust Case—control Consortium controls (www.wtccc.org.uk), the Exome Variant Server, 1000 Genomes and Complete Genomics 69 (http://www.completegenomics.com/public-data/69-Genomes/) cohorts.

## Results

**Fig. 1:**
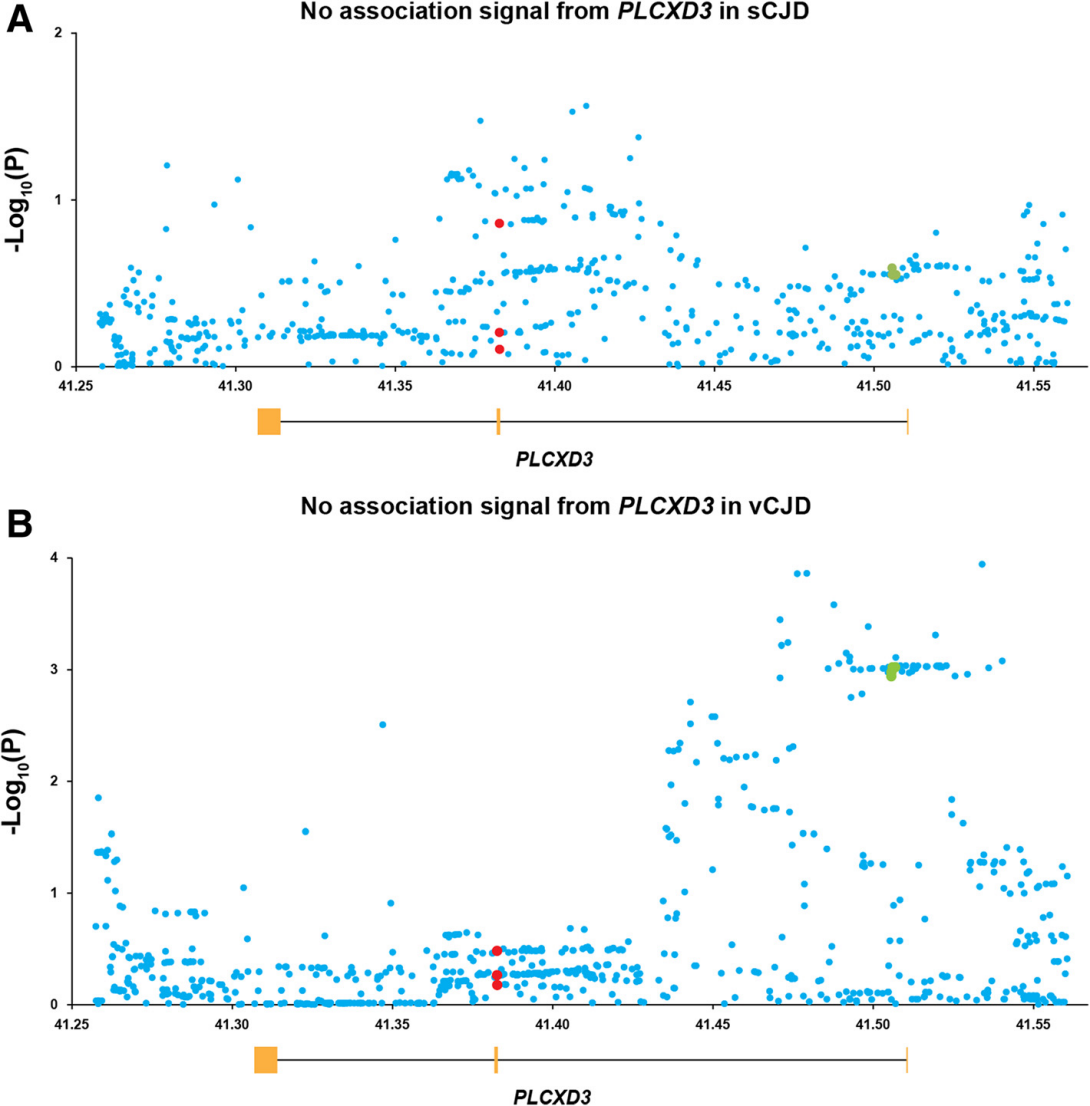
**a** There is no association signal at the *PLCXD3* locus in 2500 sCJD cases compared to 10,548 controls. (**b**) There is only a modest association signal at the *PLCXD3* locus in 129 vCJD cases compared to 5020 UK controls.−Log_10_(P) values for genotyped and imputed SNPs are plotted against their positions. *PLCXD3* SNPs found to be significant in the GWAS by Bishop et al. are indicated in green and *PLCXD3* SNPs sequenced by Bishop et al. are in red. Schematic of *PLCXD3* gene locus with exons represented in orange and intronic regions by the black line

Whilst we found modest evidence of association at SNPs in the vicinity of exon 1 in vCJD, this was far from genome-wide thresholds of significance in our sample, which largely overlaps the samples used in the original study (Fig. 1b) and therefore is not evidence of replication. Elsewhere, and importantly at the splice junction of intron 1/exon 2, we found no Hardy-Weinberg disequilibrium or association with disease for any SNPs in the GWAS data consisting of 2500 sCJD cases, 129 vCJD cases and 10,548 control individuals (Fig. 1a and b). The *PLCXD3* SNPs reported to be associated with disease risk in the GWAS by Bishop et al. [4] and the intronic SNPs found to have significantly different allele and genotype frequencies between cases and publicly available controls showed no association with disease in our study (Table 1). For example, rs688551 minor allele frequency (MAF) =0.063, odds ratio (OR) sCJD vs. control =0.92 (0.81–1.05), rs319013 MAF =0.378 OR =0.94 (0.88–1.00) and rs76547469 MAF = 0.061 OR = 0.96 (0.85–1.10). In our sCJD study, assuming we genotyped perfectly, the functional SNP has 80 % power at a MAF of 0.06 to detect an additive heterozygous effect size of 1.19 [17].

**Table 1:** *PLCXD3* SNPs identified by GWAS and reported by Bishop et al. (†) and those subsequently resequenced by Bishop et al. (‡) are shown. *P* values are given for the discovery and replication studies. SNP positions refer to GRCh37 build

SNP ID	Position	Genomic-control corrected *P*-value Bishop et al. GWAS 85 vCJD cases vs. 1481 controls	Allelic Fisher Exact *P*-value Bishop et al. Resequencing 120 vCJD cases vs. publicly available controls	Allelic Fisher Exact *P*-value Bishop et al. Resequencing 109 sCJD cases vs. publicly available controls	Frequentist additive *P*- value MRC Prion Unit GWAS 129 vCJD cases vs. 5020 UK controls	Frequentist additive *P*-value MRC Prion Unit GWAS 2500 sCJD cases vs. 10548 controls
rs3863150^†^	5:41506860	1.53 x 10^−08^	Not reported in study	Not reported in study	0.000949	0.282
rs688551^†^	5:41506080	1.53 x 10^−08^	Not reported in study	Not reported in study	0.000949	0.282
rs10075789^†^	5:41505703	1.53 x 10^−08^	Not reported in study	Not reported in study	0.00105	0.282
rs676328^†^	5:41505689	1.53 x 10^−08^	Not reported in study	Not reported in study	0.00115	0.257
rs545358^‡^	5:41382691	Not reported in study	<2.2 x 10^−16^	2.01 x 10^−5^	0.668	0.788
rs319013^‡^	5:41382681	Not reported in study	1.25 x 10^−06^	4.69 x 10^−8^	0.331	0.139
rs76547469^‡^	5:41382647	Not reported in study	0.247	0.0702	0.545	0.624

With allele discrimination probes we confirmed genotypes in 176 sCJD cases at both rs545358 (AA:AC:CC = 149:23:1) and at rs319013 (TT:TG:GG = 54:93:26) and in 119 vCJD cases at both rs545358 (AA:AC:CC = 102:17:0) and at rs319013 (TT:TG:GG = 43:58:18). Genotypes for all these cases were 100 % concordant with their imputed genotypes when scored by the threshold method. Using resequencing we further confirmed genotypes in 46 sCJD cases at rs545358 (AA:AC:CC = 35:10:1) and at rs319013 (TT:TG:GG = 12:28:6) and in 47 vCJD cases at rs545358 (AA:AC:CC = 36:11:0) and at rs319013 (TT:TG:GG = 16:23:8). Again genotypes for all these cases were 100 % concordant with their imputed genotypes in the GWAS.

Using whole exome sequencing in 376 CJD cases we found only one non-synonymous heterozygous variant in the *PLCXD3* gene in a patient with sCJD (exon2:c.C673G:p.L225V), however this variant was also present in the 1000 Genomes control population with a minor allele frequency of 0.0005. Additionally we found a separate non-synonymous heterozygous variant in *PLCXD3* in one sample in the control population which was not found in CJD cases. There was no significant association of variants in *PLCXD3* with disease in the exome sequencing data (*p* = 0.566).

## Discussion

We looked to replicate published findings at *PLCXD3* in the context of genomic studies of prion disease. We show that there is no exceptional association of variants in this gene with human prion disease. A critical step in validating GWAS signals is replication of results and we have been unable to do this in our much larger study of CJD cases which inherently has more power to detect an association [18]. A recent GWAS of 434 sCJD patients and an independent replication cohort of 1109 sCJD patients was reported but apparently did not replicate a strong signal at the *PLCXD3* locus [19]. We suggest that this gene should not be considered a priority candidate for further study as a modifier in prion disease.

There are a number of reasons which together may contribute towards the discrepancy between studies including differences in sample-size and genotyping procedures. We also considered the possibility of errors in genotyping or imputation in our study, and as a precaution against this used a second method to confirm the accuracy of our data.

We note some limitations of our own study. The sample size for vCJD cases was necessarily small and we cannot exclude the possibility of modest risk effects specific to this human prion disease. In sCJD, we can exclude all but very weak effects at the locus conferred by common SNPs, and strong effects conferred by coding variation, but we did not test for structural variation at *PLCDX3*, which was reported genome-wide elsewhere [20]. We did not study modifying effects of genetic variation on the clinical phenotype of prion disease, which can be profound [21]. We did not do functional studies of *PLCXD3*, which in our view are not warranted without substantial evidence of genetic association.

## Conclusion

Polymorphisms of *PLCXD3* are unlikely to be risk factors in human prion disease.
